# Obesity in Nursing Home Patients: Association with Common Care Problems

**DOI:** 10.3390/nu15143188

**Published:** 2023-07-18

**Authors:** Silvia Bauer, Doris Eglseer, Franziska Großschädl

**Affiliations:** Institute of Nursing Science, Medical University of Graz, Neue Stiftingtalstraße 6/P06-WEST, 8010 Graz, Austria; doris.eglseer@medunigraz.at (D.E.); franziska.grossschaedl@medunigraz.at (F.G.)

**Keywords:** obesity, nursing home, care problem, incontinence

## Abstract

(1) Background: There is not much research about obesity in nursing homes although knowledge will help us to develop customized treatment plans and prevention strategies, which may help to decrease the burden for all persons involved. The objective of conducting this study was to describe the prevalence of obesity and the association between obesity and care problems in nursing home patients. (2) Methods: This study is a secondary data analysis of data collected in an annually performed cross-sectional study called “Nursing Quality Measurement 2.0”. A standardized and tested questionnaire was used to collect data. (3) Results: In total, 1236 nursing home patients took part, and 16.7% of them were obese. The multivariate logistic regression analysis results show that urinary incontinence is significantly associated with the presence of obesity (OR 2.111). The other care problems of pressure injuries, fecal and double incontinence, physical restraints, falls, and pain were not associated with obesity. (4) Conclusions: The results indicate that, in the nursing home setting, healthcare staff should pay special attention to the patients’ nutritional status and help patients to maintain a healthy weight and prevent a loss of muscle mass and function. Conducting more studies with larger sample sizes is recommended, as this will allow for differentiation among different obesity classes.

## 1. Introduction

Obesity and being overweight are serious disorders worldwide, due to their association with chronic diseases such as diabetes mellitus type 2, coronary heart diseases, and cancer. Global estimates indicate that around 2.8 million people die annually as a result of morbid obesity. Obesity also has psychosocial consequences, such as lower quality of life and depression [1], which may also lead to discrimination, especially when combined with older age [2,3]. Obesity is a multifactorial event that usually consists of an excessively high calorie intake, the consumption of insufficient fruit and vegetables, and a lack of exercise [4]. Age, and especially older age, is a determinant for obesity [5]. However, it has also been found that younger adults with obesity experience age-related variations in immunity and biomarkers that are known to be linked to increased health risks later in life, namely chronic diseases, disability, and premature death [6].

Due to the steadily increasing life expectancy worldwide, the obesity epidemic places a heavy burden on health systems, nursing homes, and their employees. These challenges are not merely country specific. The care of nursing home patients with nutritional challenges such as obesity is a Europe-wide problem [7,8]. The admission of very old patients with severe functional impairment and an increased complexity of diseases amplifies these challenges even more [9,10].

Several researchers have described the substantial increase in the prevalence of obesity among nursing home patients [11,12]. Port Starr et al. [11] systematically reviewed the prevalence of obesity among nursing home patients, noting an increase from 14.7% in 2000 to 23.9% in 2010. Zhang et al. [12] observed that the prevalence of obesity in US nursing homes rose from 22.4% in 2005 to 28% in 2015.

The care of patients with obesity in nursing homes presents huge challenges for the nursing staff [13], and not only because patients with obesity require more nursing assistance to perform specific tasks or the use of specific equipment [8,14]. Common care problems, such as incontinence, pressure injuries, or falls, may also be more prevalent among nursing home patients with obesity, increasing the work load among nursing staff [14]. Nevertheless, the literature shows conflicting results regarding the prevalence of care problems in persons with obesity. For instance, the results on the association between incontinence and obesity and falls and fall-related injuries and obesity are contradictory; some studies have found positive associations while others have not [15,16,17,18]. Conflicting results have been reported regarding the association between obesity and functional impairment or care dependency, with some studies finding a higher prevalence of functional impairment or care dependency among patients with obesity [11,14], while other studies did not [1,8,19]. Pressure injuries seem to be more prevalent among patients with obesity than among patients without obesity [20]. This may be due to the fact that preventing pressure injuries (e.g., repositioning) in patients with obesity is particularly challenging due to the extra physical strain on the nursing staff and the lack of appropriately sized medical equipment [2]. On the other hand, it is suggested from the literature that malnutrition may be a risk factor for pressure injuries [21,22].

While the international literature contributes to our knowledge of the prevalence of obesity in nursing homes [19], most of these studies were conducted in the USA, and we know that the prevalence of obesity is much higher in the USA than in other countries [23]. This topic is of central interest to researchers worldwide, however, due to the steadily increasing prevalence of obesity among older people. Furthermore, the conflicting results reported in the literature regarding the association between common care problems and obesity indicates that further research is warranted [14]. This knowledge will help us to develop customized treatment plans and prevention strategies, which may help to decrease the burden for all persons involved in the care of nursing home patients with obesity.

Therefore, this study was conducted to answer the following research questions: What is the prevalence of obesity in nursing home patients? What is the association between obesity and common care problems in this population?

## 2. Materials and Methods

### 2.1. Research Design

This secondary data analysis included data collected in an annually performed cross-sectional study called “Nursing Quality Measurement 2.0” (LPZ Landelijke Prevalentiemeting Zorgkwaliteit study). This cross-sectional study is conducted in different health care organizations in several European countries and is performed on one specific day. The aim is to collect data on the specific care problems of pressure injuries, incontinence, malnutrition, falls, restraints, and pain [24].

### 2.2. Setting and Sample

All nursing homes in Austria with more than 50 beds were invited to participate in the study annually. Data from all nursing homes that participated from 2016–2019 (i.e., 8 November 2016, 14 November 2017, 13 November 2018, or 12 November 2019) were included in the analysis. These nursing home patients were only included after they or their legal representative gave their written informed consent. Furthermore, data from patients with no information on weight and/or height (*n* = 3) were excluded.

### 2.3. Data Collection

Data collection was performed by the nursing home staff. Therefore, a study coordinator was assigned at each participating nursing home. The coordinators were invited to a training session held by the research team members and were provided with information materials, the informed consent forms, the questionnaires, and a password for the online-data entry program at this session. Data collection was performed by a team of two trained nurses. One nurse was from the respective ward, and the other one was from another ward. The external nurse made the final decision if a disagreement arose.

The questionnaire is based on guidelines and updated annually by the international research group. The reliability and validity of the questionnaire and the included scales have been comprehensively tested and found to be good [24]. The demographic data collected included the patients’ age, sex, medical diagnoses according to ICD-10 [25], and care dependency [26].

Weight was measured directly to the nearest hundredth of a gram while the patients wore light clothes and no shoes. Height was measured in centimeters. We defined obesity by referring to the WHO classification system. A person was evaluated as obese if the BMI was higher than or equal to 30, whereby the following three classes were distinguished: class 1 (BMI 30.0–34.9 kg/m^2^), class 2 (BMI 35.0–39.9 kg/m^2^), and class 3 (BMI ≥ 40.0 kg/m^2^) [4].

The care dependency scale (CDS) was used to measure care dependency. It is a 15-item scale and the scores for each item range from 1 (completely dependent) to 5 (completely independent). The higher the sum score, the lower the care dependency [26].

Pressure injuries were categorized according to the NPIAP, the EPUAP, and the PPPIA [27]. Incontinence was categorized as an involuntary loss of only urine (urinary incontinence) or only fecal material (fecal incontinence) or both (double incontinence) [28]. A fall is an incident where the patient falls to the ground or lower level unintentionally [29]. Physical restraints are defined as all measures restricting human rights and freedom of movement. That includes all restrictions of personal mobility, such as observation, isolation, manual restraints, and the use of psychological measures [30]. Pain was defined as an unpleasant sensory and emotional experience that is associated with actual or potential tissue damage or can be described in terms of such damage [31].

### 2.4. Ethical Considerations

Prior to their inclusion in the study, a written informed consent form had to be signed by all participating patients or their legal representatives. Approval of the study protocol was obtained from the local ethical committee (20-192 ex 08/09) and the study conforms to recognized standards and the Declaration of Helsinki.

### 2.5. Data Analysis

The data analysis was performed using IBM SPSS Statistics 26 for Windows (IBM). All variables were analyzed descriptively. The chi-squared test and the Mann–Whitney U test were used to identify differences between the groups. A stepwise multivariate logistic regression analysis was performed with obesity as the dependent variable. No multicollinearity was presumed if the variance inflation factors were below four [32,33]. In addition, 95% confidence intervals for the odds ratios were calculated, and the Hosmer–Lemeshow test was performed to assess the goodness of fit of the final model. *p*-values lower than 0.05 were considered as statistically significant.

## 3. Results

In total, 1528 nursing home patients were eligible to participate in the study, and 1239 (81.1%) participated in the surveys. The main reasons certain patients did not take part in the survey included refusal to participate (43.9%) and cognitive impairment (23.9%). Three patients lacked BMI data and, therefore, were excluded, resulting in a study population of 1236 nursing home patients.

**Figure 1:**
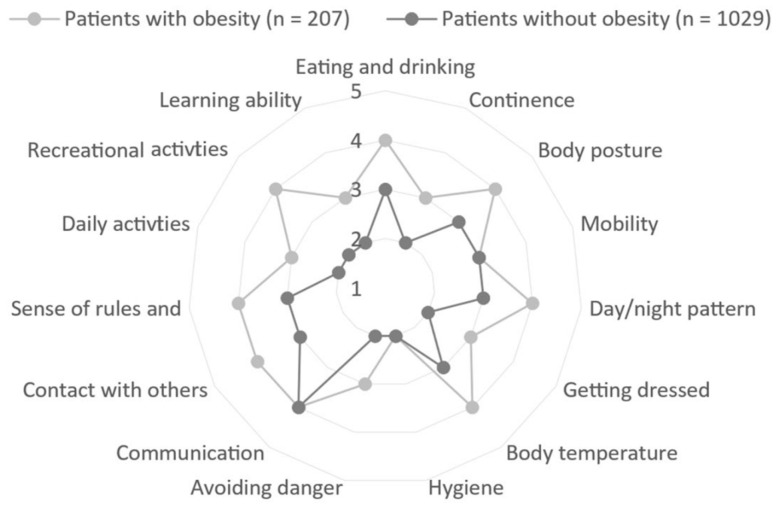
Median item values of the CDS in the comparison between patients with and without obesity. The care dependency scale (CDS) was used to measure care dependency; possible scores range between 15 and 75, with lower scores indicating higher care dependency.

Within this sample, 16.7% of the patients were obese. Of the patients identified as obese (*n* = 207), 70.5% belonged to obesity class 1, 19.8% belonged to obesity class 2, and 9.7% belonged to obesity class 3. In total, 71.4% of the patients were female (Table 1). Patients without obesity were significantly older, and the care dependency of these patients was higher than patients with obesity. The higher care dependency of patients without obesity is true for all items except mobility, hygiene, and communication, whereby the median was the same for both groups (Figure 1).

We found a significant difference in the prevalence of urinary incontinence between patients with and without obesity, with the results showing that patients with obesity were more often affected as compared to patients without obesity (Table 2). Double incontinence was statistically significantly more prevalent in patients without obesity. Physical restraints and fall-related injuries were also significantly more prevalent among patients without obesity.

In the multivariate regression analysis, several variables were found to be statistically significantly associated with obesity (Table 3). Diabetes was most strongly associated with obesity (OR 2.590), followed by urinary incontinence (OR 2.111). The risk of obesity negatively correlated with both age and care dependency: the older the patient was and the more care dependent the patient was, the lower their risk of obesity.

## 4. Discussion

This study was carried out to describe the prevalence of obesity and the associations between obesity and common care problems observed among nursing home patients. The results show that 16.7% of the patients were obese. The multivariate logistic regression analysis shows that urinary incontinence was significantly associated with the presence of obesity while the other care problems were not. Age and care dependency were found to be negatively associated with obesity.

In our study sample, the prevalence of obesity in this population was lower than the prevalence reported in other studies. However, most of these studies were conducted in the USA, where the prevalence of obesity is known to be remarkably higher than in European countries [11,12]. We found that patients with obesity are younger and less care dependent than patients without obesity.

Our study results indicate that urinary incontinence is strongly associated with obesity. This finding is in line with those of other studies [16,17]. Nevertheless, the reasons behind and the association between these two topics are highly complex. One possible explanation for this finding is that a high quantity of abdominal fat places increased pressure on the pelvis (inter-abdominal and intravesical), causing the patient to lose urine involuntarily [34,35]. Therefore, abdominal obesity seems to be more strongly associated with urinary incontinence than general obesity (i.e., only a high BMI). Park and Baek [34] showed that women with a normal BMI but a high waist circumference were more likely to report symptoms of urinary incontinence than overweight women (BMI ≥ 25) alone. This hypothesis has been supported by the results of another study conducted in older adults [35]. Furthermore, obesity may lead to disturbances in oxidative metabolism and insulin resistance. These developments can damage vessels in the pelvic floor, increasing the risk of developing incontinence [35]. There are also other contributing factors, like diabetes, sleep apnea, limited mobility, childbirth, and medication like diuretics. These factors should be addressed properly. Weight loss may not be the right solution to urinary incontinence for every patient, and its effect is not yet proven [36].

A patient that is both urinary incontinent and obese has an increased need for assistance in performing care tasks, such as toileting and maintaining personal hygiene [7,13,14]. A failure to provide adequate and timely assistance with toileting (e.g., due to staff restraints) may also lead to incontinence episodes in persons with obesity and, consequently, increase the risk of incontinence-associated dermatitis [17]. Henderson et al. [37] also showed that urinary incontinence and skin infections are common comorbidities associated with obesity. Although there are some sources in the literature suggesting that a controlled loss of fat mass in patients with obesity may help to reduce the prevalence of incontinence [15], this should be interpreted with caution since a weight loss intervention may lead to a loss of muscle mass, which should by all means be avoided in older patients [36,38].

We found that most of the care problems we measured in our study were not associated with the presence of obesity. This supports the assumption that the “obesity paradox” may also be present in nursing home patients with regard to care problems. The “obesity paradox” suggests that patients with obesity have a survival advantage over patients without obesity [39]. Our study results indicate that nursing home patients with obesity may also have an advantage with regard to how often they experience care problems, such as falls, pressure injuries, or care dependency. One exception seems to be urinary incontinence; whereby abdominal fat accumulation is one major risk factor.

While some older people experience problems stemming from excess weight such as obesity, other older people—especially very old persons—experience problems associated with being underweight: malnutrition. A systematic review and meta-analysis was conducted to assess the prevalence of malnutrition in nursing homes and revealed that this affects up to 30% of the patients in this setting [40], with up to 44% of the patients identified as at risk of developing malnutrition [41]. In 2018, the results of an Austrian annual measurement showed that 42.6% of the nursing home patients in the participating nursing homes were affected by malnutrition or malnutrition risk [42]. Our results suggest that malnutrition seems to be an even bigger problem in the nursing home setting in Austria than obesity which is associated with serious side effects, such as a higher risk of falls, pressure injuries, care dependency, and impaired mobility [21,34,43].

To our knowledge, this is the first study in which care dependency and common care problems and their association with obesity for a large sample of 1236 nursing home patients are described. The BMI of the patients was measured and not self-reported, which increases the data validity [44]. However, there are some limitations. The interpretation of associations between obesity and care problems or general health status is complex because obesity can also be associated with sarcopenia, which is the loss of muscle mass and function, leading to sarcopenic obesity [45]. We did not collect data regarding muscle mass and function, which limits the results. Additionally, the convenience sampling method that was used leads to results that cannot be generalized. The low number of obese persons categorized into obesity classes 2 and 3 did not allow the data to be stratified for the different obesity classes. If it had been possible to compare the prevalence of care problems between patients in different obesity classes, we could have gained an even better insight into the topic.

## 5. Conclusions

The study shows that patients with obesity differ from those without obesity in terms of age and care dependency. Most of the care problems are not more prevalent in nursing home patients with obesity than in nursing home patients without obesity, except for urinary incontinence. In the nursing home setting, health care staff should pay special attention to the patients’ nutritional status. However, the prevention of a loss of muscle mass and function (sarcopenia) is of utmost importance. This loss of muscle mass and function is often masked by the parallel occurrence of obesity and sarcopenia, namely sarcopenic obesity. Nurses caring for older people should provide support that helps the patient maintain their normal, healthy weight. This is especially true for patients with urinary incontinence because urinary incontinence in patients with obesity can have many negative consequences (e.g., skin infections). Obesity adds to the burden of care for nursing home staff [8], but the answer to this problem is in the responsibility of the respective care organizations and should not negatively impact a patient’s health or their quality of life. Consequently, obesity should also be discussed in nursing education, enabling nurses to offer the affected people the best possible care and to counteract discrimination against patients with obesity [2]. So far, nursing researchers in Europe have paid little attention to obesity. Therefore, we recommend that more nursing research be conducted on obesity and its relation to specific care problems, including larger samples. This will allow researchers to differentiate between the different obesity classes and potentially confirm our study’s results.

## Figures and Tables

**Table 1:** Sample characteristics.

	Total Patients (*N* = 1236)	Patients with Obesity (*n* = 207)	Patients without Obesity (*n* = 1029)	*p*-Value *
Female sex (in %)	71.4	67.1	72.2	0.12
Age median (range) (in years)	86 (12)	82 (14)	87 (11)	<0.001
Care dependency median (range) ^†^	44 (29)	53 (23)	41 (30)	<0.001
BMI median (range) in kg/m^2^	24.5 (6.6)	33.2 (3.7)	23.5 (5.2)	<0.001
Medical diagnosis (in %)				
Diseases of the circulatory system	71.7	73.9	71.2	0.44
Dementia	54.9	42.0	57.4	<0.001
Diseases of the musculoskeletal system	49.4	49.3	49.4	0.98
Mental diseases	42.1	45.4	41.4	0.29
Diseases of the genitourinary system	32.7	33.8	32.5	0.70
Diseases of the digestive system	31.2	34.3	30.6	0.30
Metabolic diseases	28.7	35.7	27.3	0.014
Diseases of the eye or ear	22.8	21.3	23.1	0.56
Diseases of the nervous system	21.0	17.9	21.6	0.23
Diabetes	20.2	35.3	17.2	<0.001
Diseases of the respiratory system	18.9	20.3	18.7	0.59
Stroke	15.8	16.4	15.6	0.78
Diseases of the blood	15.2	13.5	15.5	0.46
Other diseases	11.7	13.0	11.5	0.52
Diseases of the skin	11.4	14.5	10.8	0.13
Other factors influencing health use	9.3	8.2	9.5	0.55
Cancer	8.8	5.8	9.4	0.09
Addiction	5.6	8.2	5.1	0.07
Infectious diseases	1.6	1.9	1.6	0.70
Number of comorbidities median (range)	5 (3)	5 (3)	5 (3)	0.284

* *p*-value between patients with and without obesity. Χ2 test for categorical variables; Mann–Whitney U test for non-parametric variables. ^†^ The care dependency scale (CDS) was used to measure care dependency; possible scores range between 15 and 75, with lower scores indicating higher care dependency.

**Table 2:** Prevalence (confidence intervals).

	Total Patients (*N* = 1236)	Patients with Obesity (*n* = 207)	Patients without Obesity (*n* = 1029)	*p*-Value *
Pressure injury	4.6 [3.6–5.8]	3.4 [0.0–5.1]	4.9 [3.7–6.6]	0.35
Urinary incontinence ^#^	37.4 [35.1–39.7]	47.2 [40.9–53.8]	35.5 [32.6–38.0]	0.002
Fecal incontinence	3.0 [2.2–4.0]	3.9 [1.4–6.8]	2.8 [1.9–3.8]	0.42
Double incontinence	34.3 [31.8–36.9]	16.9 [12.3–21.5]	37.7 [34.8–40.6]	<0.001
Physical restraints	11.2 [9.4–12.9]	5.9 [2.9–9.3]	12.2 [10.4–14.1]	0.009
Falls ^†^	17.0 [14.8–19.3]	11.6 [7.5–15.6]	18.0 [15.5–20.6]	0.06
Fall-related injuries ^†^	8.9 [7.2–10.4]	4.1 [2.0–6.8]	9.8 [7.9–11.9]	0.026
Pain (in general)	40.1 [37.6–42.6]	43.0 [36.2–49.8]	39.6 [36.7–42.3]	0.36
Acute pain	5.7 [4.4–6.9]	3.9 [1.4–6.8]	6.0 [4.7–7.4]	0.22
Chronic pain	34.5 [32.0–37.0]	39.1 [32.4–45.9]	33.5 [30.9–36.2]	0.12

* *p*-value between patients with and without obesity. Χ2 test for categorical variables; Mann–Whitney U test for non-parametric variables. ^#^ *n* = 1192 because patients with a catheter were excluded. ^†^ *n* = 914 because of missing data.

**Table 3:** Univariate and multivariate logistic regression with obesity as the outcome (*N* = 1236).

	Univariate		Multivariate	
	*p*-value	OR [95% CI]	*p*-value	OR [95% CI]
Sex	0.14	0.787 (0.571–1.084)		
Age	<0.001	0.962 (0.949–0.976)	<0.001	0.938 (0.918–0.958)
Number of comorbidities	0.16	1.051 (0.981–1.127)		
Care dependency (sum score) *	<0.001	1.026 (1.017–1.035)	<0.001	1.033 (1.020–1.047)
Pressure injuries	0.36	0.685 (0.306–1.532)		
Falls	0.06	0.596 (0.348–1.021)		
Fall-related injuries	0.031	0.393 (0.168–0.920)	0.06	0.392 (0.148–1.040)
Physical restraints	0.010	0.449 (0.243–0.828)		
Urinary incontinence	0.002	1.622 (1.190–2.211)	<0.001	2.111 (1.416–3.146)
Fecal incontinence	0.42	1.386 (0.625–3.077)		
Double incontinence	<0.001	0.336 (0.22–0.500)		
Pain (in general)	0.36	1.153 (0.852–1.559)		
Diabetes	<0.001	2.622 (1.889–3.640)	<0.001	2.590 (1.680–3.991)
Dementia	<0.001	0.537 (0.397–0.727)		
Metabolic diseases	0.015	1.481 (1.080–2.031)		

Cox and Snell’s R2 0.113; Nagelkerke’s R2 0.194; Hosmer–Lemeshow test Χ2 3.953; *df* = 8; *p* = 0.861. * The care dependency scale (CDS) was used to measure care dependency; possible scores range between 15 and 75, with lower scores indicating higher care dependency. No is always the reference category.

## Data Availability

The data presented in this study are available from the corresponding author upon request. The data are not publicly available due to legal reasons.
